# Dissecting the Polyhydroxyalkanoate-Binding Domain of the PhaF Phasin: Rational Design of a Minimized Affinity Tag

**DOI:** 10.1128/AEM.00570-20

**Published:** 2020-06-02

**Authors:** Aranzazu Mato, Francisco G. Blanco, Beatriz Maestro, Jesús M. Sanz, Jesús Pérez-Gil, M. Auxiliadora Prieto

**Affiliations:** a Polymer Biotechnology Group, Microbial and Plant Biotechnology Department, Centro de Investigaciones Biológicas Margarita Salas-CSIC, Madrid, Spain; b Interdisciplinary Platform for Sustainable Plastics towards a Circular Economy‐Spanish National Research Council (SusPlast‐CSIC), Madrid, Spain; c Host-Parasite Interplay in Pneumococcal Infection Group, Microbial and Plant Biotechnology Department, Centro de Investigaciones Biológicas Margarita Salas-CSIC, Madrid, Spain; d Centro de Investigación Biomédica en Red de Enfermedades Respiratorias (CIBERES), Madrid, Spain; e Biochemical and Molecular Biology Department, Facultad de Ciencias Biológicas, Universidad Complutense de Madrid, Madrid, Spain; University of Tartu

**Keywords:** biotechnology, functionalization, phasins, polyhydroxyalkanoates, *Pseudomonas putida*

## Abstract

Phasin PhaF from Pseudomonas putida consists of a modular protein whose N-terminal domain (BioF) has been demonstrated to be responsible for binding to the polyhydroxyalkanoate (PHA) granule. BioF has been exploited for biotechnological purposes as an affinity tag in the functionalization of PHA beads with fusion proteins both *in vivo* and *in vitro*. The structural model of this domain suggests an amphipathic α-helical conformation with the hydrophobic residues facing the PHA granule. In this work, we analyzed the mean hydrophobicity and the hydrophobic moment of the native BioF tag to rationally design shorter versions that maintain affinity for the granule. Hybrid proteins containing the green fluorescent protein (GFP) fused to the BioF derivatives were studied for *in vivo* localization on PHA, stability on the surface of the PHA granule against pH, temperature, and ionic strength, and their possible influence on PHA synthesis. Based on the results obtained, a minimized BioF tag for PHA functionalization has been proposed (MinP) that retains similar binding properties but possesses an attractive biotechnological potential derived from its reduced size. The MinP tag was further validated by analyzing the functionality and stability of the fusion proteins MinP–β-galactosidase and MinP-CueO from Escherichia coli.

**IMPORTANCE** Polyhydroxyalkanoates (PHAs) are biocompatible, nontoxic, and biodegradable biopolymers with exceptional applications in the industrial and medical fields. The complex structure of the PHA granule can be exploited as a toolbox to display molecules of interest on their surface. Phasins, the most abundant group of proteins on the granule, have been employed as anchoring tags to obtain functionalized PHA beads for high-affinity bioseparation, enzyme immobilization, diagnostics, or cell targeting. Here, a shorter module based on the previously designed BioF tag has been demonstrated to maintain the affinity for the PHA granule, with higher stability and similar functionalization efficiency. The use of a 67% shorter peptide, which maintains the binding properties of the entire protein, constitutes an advantage for the immobilization of recombinant proteins on the PHA surface both *in vitro* and *in vivo*.

## Abstract (precis)

Polyhydroxyalkanoates (PHAs) are biocompatible, nontoxic, and biodegradable biopolymers with exceptional applications in the industrial and medical fields. The complex structure of the PHA granule can be exploited as a toolbox to display molecules of interest on their surface. Phasins, the most abundant group of proteins on the granule, have been employed as anchoring tags to obtain functionalized PHA beads for high-affinity bioseparation, enzyme immobilization, diagnostics, or cell targeting. Here, a shorter module based on the previously designed BioF tag has been demonstrated to maintain the affinity for the PHA granule, with higher stability and similar functionalization efficiency. The use of a 67% shorter peptide, which maintains the binding properties of the entire protein, constitutes an advantage for the immobilization of recombinant proteins on the PHA surface both *in vitro* and *in vivo*.

## INTRODUCTION

The display of functional proteins on solid supports constitutes an important tool for many industrial and biomedical purposes (1). In this vein, nanoparticles functionalized with peptides and proteins may be widely employed for therapeutic applications, acting as drug carriers, antitumor and bactericidal drugs, or cellular targeting moieties (2). Strategies for protein immobilization vary depending on the matrixes employed and the final application, and they may include nonspecific adsorption, chemical cross-linking, or the use of affinity tags (3).

**FIG 1:**
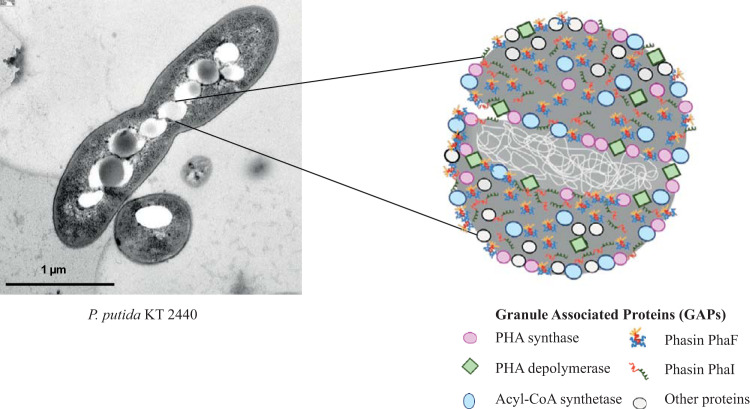
PHA production and granule structure. (Left) Transmission electron microscopy (TEM) image of P. putida KT2440 growing under PHA-producing conditions. Granules accumulated as hydrophobic inclusions inside the cell. (Right) Schematic representation of the PHA granule from P. putida KT2440. The PHA is surrounded by a layer of proteins, called granule-associated proteins (GAPs), such as PHA polymerase, depolymerase, and phasins. Acyl-CoA, acyl-coenzyme A.

The biodegradable and biocompatible polyhydroxyalkanoates (PHAs) are bacterial storage polyesters that are gaining much attention as highly tunable materials for the development of a variety of biomedical and industrial devices. This is mainly due to the structural versatility that can be obtained by bacterial fermentation and postbiosynthetic modifications (4). These polymers are synthesized as hydrophobic inclusions surrounded by a layer of proteins involved in the PHA metabolism, called granule-associated proteins (GAPs). Among them, the most remarkable are PHA polymerases and depolymerases, as well as phasins, which are amphipathic proteins coating the PHA granule (Fig. 1) (5). The complex architecture of PHA granules offers a toolbox to display molecules of interest on their surfaces (6). GAPs have been used as anchoring tags to immobilize value-added proteins on the surface of PHA materials. Depending on the target application, functionalization by using GAP fusion proteins may be achieved *in vivo* (simultaneously with PHA production in the bacterial cytoplasm) or *in vitro* (utilizing endotoxin-free PHA purified after biotechnological production) (7). The first strategy led to GAP fusion proteins immobilized on the PHA surface simultaneously with their synthesis. The second approach requires the extraction of the polyester, its conversion into nanoparticles or films, and the subsequent *in vitro* immobilization of the GAP fusion. This strategy is convenient for biomedical purposes that require endotoxin-free PHA materials and a control of the amount of the immobilized protein. Among GAPs, phasins constitute the most abundant group of proteins covering the granule (8, 9) and have been widely used as anchoring tags to functionalize PHA supports for a variety of applications, such as affinity bioseparation (10), protein purification (11–13), enzyme immobilization (14–16), protein delivery to natural environments (17), diagnostics (18, 19), cell targeting (20–24), and antimicrobials (25).

PhaF from Pseudomonas putida is a prototypic and well-characterized phasin (26, 27). It is a partially intrinsically disordered protein consisting of two domains connected by a leucine zipper motif that is responsible for its oligomerization (28, 29). The 142-amino-acid (aa) BioF module, containing the N-terminal and leucine zipper sequences, has been extensively demonstrated to bind to PHA granules (17, 30, 31). In addition, the BioF domain binds not only to PHA materials *in vitro* but also to other hydrophobic-hydrophilic interfaces, such as those containing phospholipids, making it a versatile tag to combine with a variety of hydrophobic supports (14, 32). One of the drawbacks of applying the BioF tag for biomedical applications is its size, as a smaller peptide would be more desirable to reduce potential interferences with correct folding and activity of the tagged protein of interest (33).

Based on the predicted structural model of PhaF that supports a conformation-dependent binding of the BioF fragment to the PHA granule (28), we have explored the ability of reduced segments of BioF to maintain the capacity to bind PHA, with the aim of finding a shorter, yet fully functional, BioF segment to be used as an efficient affinity tag for recombinant protein immobilization.

## RESULTS

### BioF-based tag design based on structure prediction.

**FIG 2:**
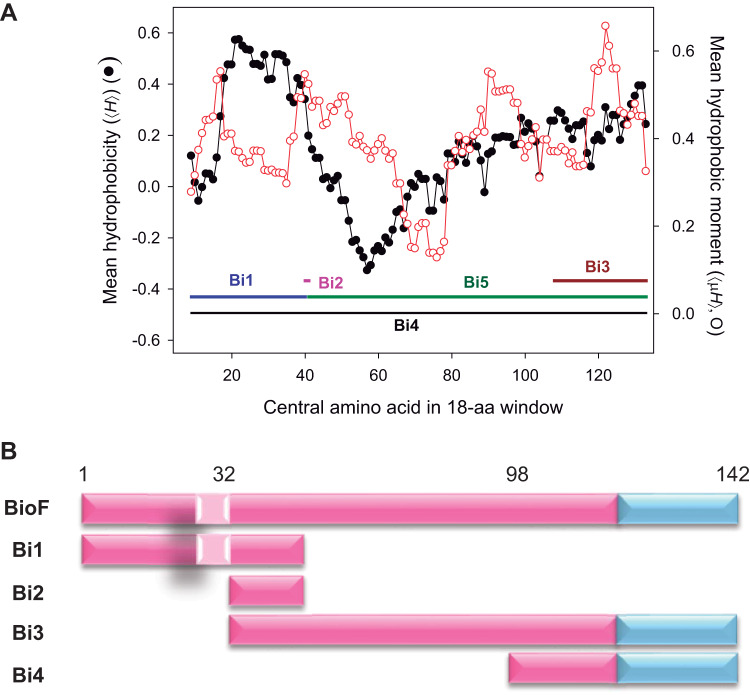
Evaluation of the structural aspects of the N-terminal domain of phasin PhaF (BioF) and design of a set of BioF-based fragments. (A) Analysis of mean hydrophobicity (<*H*>) and mean hydrophobic moment (<μ*H*>) of predicted α-helical stretches within the PHA binding and leucine zipper motifs of PhaF. Calculations were performed with the HeliQuest utilities using a sequence window size of 18 aa (46). (B) Schematic representation of the battery of BioF-based peptides designed. BioF amino acid residue positions are indicated above, pink corresponds to the N-terminal domain of PhaF, light pink corresponds to the conserved highly hydrophobic motif (WLAGLGI), and blue corresponds to the central leucine zipper motif.

The N-terminal domain of PhaF (N-PhaF) is predicted to form a long, largely amphipathic α-helix that interacts with PHA through hydrophobic interactions while exposing the hydrophilic side to solvent or the cytoplasmic fraction of the cell (27). A HeliQuest analysis of the N-PhaF sequence shows the mean hydrophobicity (<*H*>) and the amphipathicity (i.e., hydrophobic moment, or <μ*H*>) of all possible 18-aa α-helical stretches within this domain (Fig. 2A) and reveals fluctuations of both properties throughout the domain. Interestingly, the region consisting of residues 33 to 49 simultaneously exhibits relatively high values of both <*H*> and <μ*H*>, while residues 58 to 91 displayed the lowest values of <*H*> and <μ*H*>. The latter region has been suggested to be natively unfolded in solution when not complexed with PHA (Fig. 2A) (28). To further determine the contributions of different segments of N-PhaF to the binding to PHA, we designed shortened versions of BioF (Fig. 2B), differing in polarity, amphipathicity, and length, namely, Bi1, Bi2, Bi3, and Bi4. The Bi1 segment contains the N-terminal region and the most hydrophobic stretches, including the residue 26 to 32 subsequence (WLAGLGI) that is conserved in all PhaF-like phasin proteins and has been proposed to play a fundamental role in the interaction of phasins with PHA granules (30). Bi2 contains the residue 33 to 49 subsequence mentioned above, whereas Bi3 starts with the Bi2 segment and extends to contain the central leucine zipper region. Finally, the Bi4 segment was designed on a different basis, as it only contains the predicted leucine zipper motif involved in PhaF oligomerization along with a short adjacent sequence (28, 29).

### *In vivo* localization of BioF-based fragments in P. putida KT2440.

**FIG 3:**
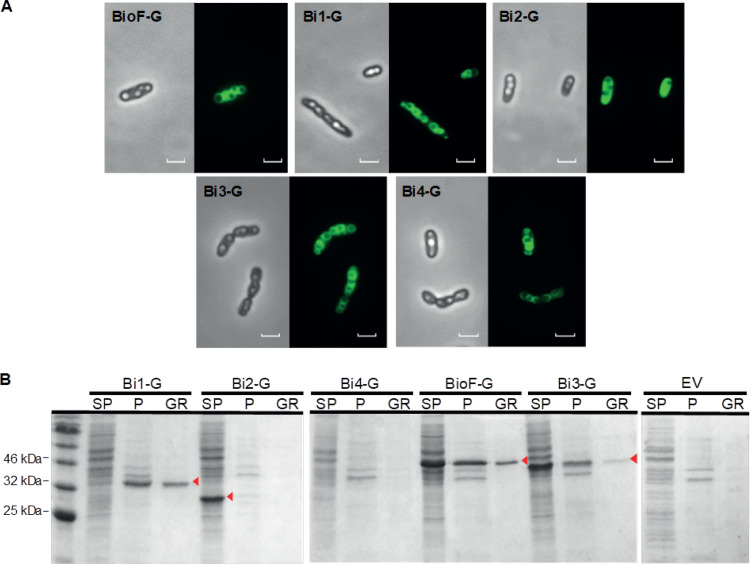
Localization of the different Bi segment-GFP fusion proteins in P. putida KT2440 after 24 h of growth in 0.1 N M63 plus 15 mM octanoate, induced at an OD_600_ of 0.8 with 1 mM 3-MB. BioF-G, P. putida KT2440(pSP1BioF-G); Bi1-G, P. putida KT2440(pSP1Bi1-G); Bi2-G, P. putida KT2440(pSP1Bi2-G); Bi3-G, P. putida KT2440(pSP1Bi3-G); Bi4, P. putida KT2440(pSP1Bi4-G). (A) *In vivo* localization of BioF-based tag segments fused to GFP by epifluorescence and phase-contrast microscopy. Scale bars, 2 μm. (B) SDS-PAGE stained with Coomassie brilliant blue. The lanes contain the fractions isolated (SP, crude extract supernatant; P, crude extract pellet; GR, isolated PHA granules). EV, P. putida KT2440(pSP1) empty vector. The volume loaded in each well was 15 μl. Red arrows indicate the predicted molecular weights of the recombinant proteins.

To study the *in vivo* PHA binding properties of the different Bi segments, we designed several green fluorescent protein (GFP) fusion constructs, which allowed for the determination of their intracellular localization. Each construct (named Bi1-G, Bi2-G, Bi3-G, Bi4-G, and BioF-G) was inserted in the pSEVA238 plasmid under the control of the XylS/P*m* regulator/promoter system (see Table 3) (34) and introduced into P. putida KT2440. Cultures were grown in 0.1 N M63 plus 15 mM octanoate to favor PHA accumulation, and the production of the corresponding recombinant protein was induced at an optical density at 600 nm (OD_600_) of 0.8 by the addition of 1 mM 3-methylbenzoate (3-MB). After 24 h of growth, cells were observed through epifluorescence microscopy (Fig. 3A). A common phenotype was detected in the strains containing BioF-G, Bi1-G, Bi3-G, and Bi4-G fusions, which showed an enriched ring of fluorescence surrounding the PHA granules (Fig. 3A). This pattern suggests the localization of these fusion proteins on the surface of the granules. A different phenotype could be observed in cells containing the shortest segment, Bi2-G, in which the protein was observed to be homogeneously distributed throughout the cytoplasm. This localization technique was complemented by glycerol gradient separation and isolation of PHA granules from cells producing the Bi-G segments (see Materials and Methods for details). Supernatant and pellet fractions of crude extracts and purified granules were separated by SDS-PAGE (Fig. 3B). Bi1-G, Bi3-G, and BioF-G proteins were detected in the granule fraction, while Bi2-G was only detected in the supernatant of the crude extract, validating the results obtained microscopically. Some Bi3-G and BioF-G proteins were also detected in the supernatant fraction of the crude extract. The inability to detect Bi4-G in any fraction by SDS-PAGE suggests that this polypeptide is produced at a low level, is unstable, or forms aggregates that cannot enter the gel. Due to these possibilities, the Bi4 segment was discarded for further studies.

### *In vivo* localization of BioF-based fragments in a P. putida KT2440 Δ*pha* strain containing PhaC1 synthase.

Phasins covering the PHA granule have been demonstrated to interact with other granule-associated proteins (GAPs) through the leucine zipper motif (29). To avoid potential interference by the presence of other GAPs on the surface of the PHA granule, a P. putida KT2440 mutant lacking the *pha* gene cluster was employed as a host to introduce solely the *phaC1* synthase gene, sufficient to produce a low level of PHA. *phaC1* expression was under the control of a weak constitutive promoter and integrated in the chromosome via the pTn*7*-M transposon system. The resulting strain was named the Δ*pha+C1* strain and was employed to confirm the ability of the Bi fragments to maintain their affinity for the PHA granule in the absence of other phasins and GAPs.

**FIG 4:**
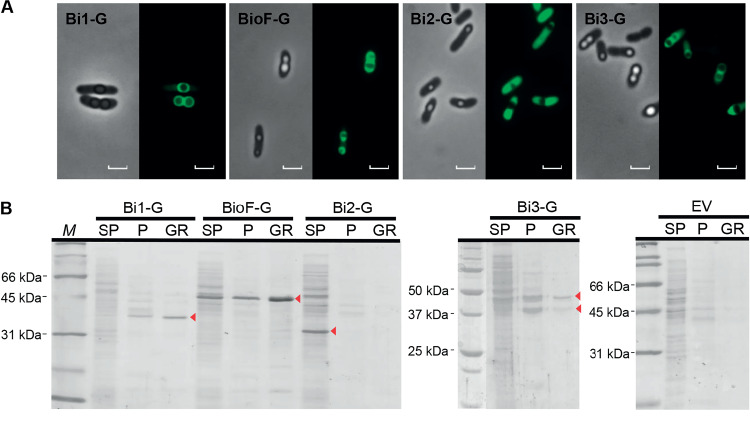
Localization of the different Bi segment-GFP fusion proteins in P. putida KT2440 *Δpha+C1* after 24 h of growth in 0.1 N M63 plus 15 mM octanoate, induced at an OD_600_ of 0.8 with 1 mM 3-MB. Bi1-G, P. putida KT2440 *Δpha*+*C1*(pSP1Bi1-G); Bi2-G, P. putida KT2440 *Δpha*+*C1*(pSP1Bi2-G); BioF-G, P. putida KT2440 *Δpha*+*C1*(pSP1BioF-G); Bi3-G, P. putida KT2440 *Δpha*+*C1*(pSP1Bi3-G). (A) *In vivo* localization of BioF-based tag segments fused to GFP by epifluorescence and phase-contrast microscopy. Scale bars, 2 μm. (B) SDS-PAGE stained with Coomassie brilliant blue. The lanes contain the fractions isolated (SP, crude extract supernatant; P, crude extract pellet; GR, isolated PHA granules; M, molecular size marker). EV, P. putida KT2440 *Δpha*+*C1*(pSP1) empty vector. The volume loaded in each well was 15 μl. Red arrows indicate the predicted molecular weights of the recombinant proteins.

The plasmids coding for the different Bi segment-GFP fusion constructs were introduced into the Δ*pha+C1* strain and assessed for localization by epifluorescence microscopy. The Bi1-G protein was observed to localize in rings surrounding the PHA granules as the control BioF-G protein (Fig. 4A), and both proteins were detected in the pellet and PHA granule fractions, with the additional presence of BioF-G in the supernatant portion (Fig. 4B). Bi3-G showed a localization pattern similar to that of BioF-G, with some fluorescence observed in the cytoplasm away from the granule surface (Fig. 4A). These results are in agreement with the fractionation studies (Fig. 4B), as some Bi3-G and BioF-G proteins were detected in the soluble fraction. In addition, some degradation of Bi3-G seemed to occur, indicated as double red arrows in Fig. 4B. In the case of Bi2-G, the fluorescence was found distributed inside the whole cell, suggesting again that this segment is unable to bind to PHA granules (Fig. 4A). This hypothesis is further confirmed by the lack of granule association in the fractionation experiment (Fig. 4B).

### Stability of BioF-based structures on the PHA granule.

Previous work had demonstrated the stability of the adsorption of BioF fusion proteins to isolated PHA granules through the application of a panel of detergents (14, 30). One of the most effective detergents for disrupting the BioF-PHA granule interaction is Triton X-100. Thus, we used this detergent to assess the binding strength of the three efficient adsorption variants to PHA, Bi1-G, Bi3-G, and BioF-G. PHA granules isolated from a 50-ml culture of P. putida KT2440 Δ*pha+C1* strains were resuspended in different concentrations of Triton X-100 and incubated for 2 h at room temperature. After the treatment, the supernatant and pellet fractions of the suspension were separated, and the protein released from the granules was evaluated by SDS-PAGE (see Fig. S1 in the supplemental material).

**FIG 5:**
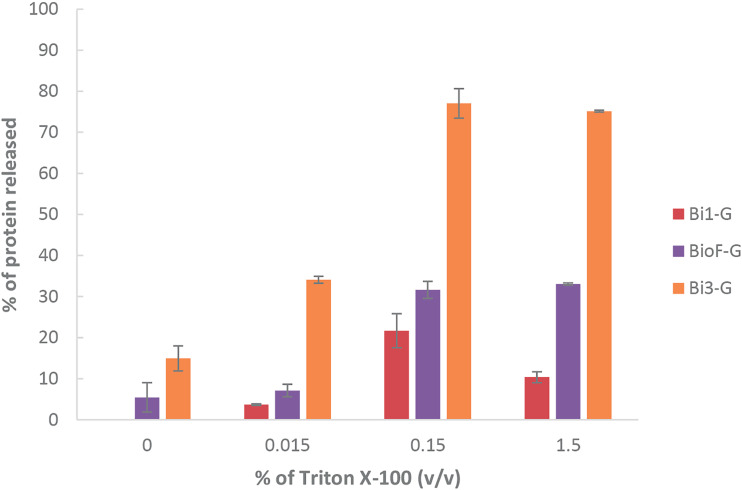
Percentage of protein released from isolated PHA granules of P. putida KT2440 Δ*pha+C1*(pSP1Bi1-G), P. putida KT2440 Δ*pha+C1*(pSP1BioF-G), and P. putida KT2440 Δ*pha+C1*(pSP1Bi3-G) after treatment for 2 h with various concentrations of Triton X-100 at room temperature and separating released (supernatant) and PHA-bound (pellet) protein. Percentages refer to the ratio between the soluble fraction and the total amount of protein (sum of soluble and insoluble fractions) using ImageJ to quantitate band intensities on Coomassie-stained SDS-PAGE. Error bars represent the standard deviations (SD) from three biological replicates and two technical replicates each.

The gel band intensities were used to calculate the percentage of Bi segment protein released from the PHA granules (Fig. 5). The data indicate that Bi3-G presents the weakest interaction with the PHA granules, even in the absence of detergent. In contrast, the protein Bi1-G was not released in the absence of detergent, and, in any case, it remained ca. 90% adsorbed on the granule even at 1.5% Triton X-100, surpassing the native BioF-G. This indicates that Bi1 displays an affinity to PHA granules similar to or even higher than that of BioF; therefore, we focused our efforts on further evaluating this segment as a potential tag for biotechnological uses.

**FIG 6:**
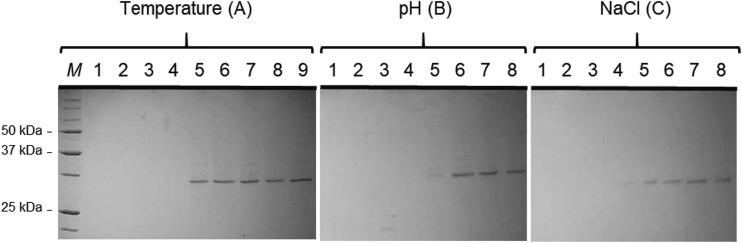
Coomassie-stained SDS-PAGE of PHA granules extracted from P. putida KT2440 Δ*pha+C1* producing Bi1-G. The stability of Bi1-G interacting with PHA granules was assessed by exposure to a range of temperatures (A), pHs (B), and ionic strengths (C) for 2 h. (A) Lanes 1 to 4, released soluble fraction at −20°C, 4°C, 37°C, and 60°C, respectively. Lanes 5 to 8, PHA granule retained protein fraction after treatment at −20°C, 4°C, 37°C, and 60°C, respectively; lane 9, untreated isolated granules. (B) Lanes 1 to 4, released soluble fraction after treatment with pH 3.0, 5.0, 7.0, and 9.0, respectively. Lanes 5 to 8, PHA granule retained protein fraction after treatment at pH 3.0, 5.0, 7.0, and 9.0, respectively. (C) Lanes 1 to 4, released soluble fraction after treatment with 0, 10, 100, and 1,000 mM NaCl, respectively. Lanes 5 to 8, PHA granule retained protein fraction after treatment with 0, 10, 100, and 1,000 mM NaCl, respectively. Volumes loaded correspond to 15 μl of the soluble and insoluble fractions obtained after the treatment of the granules.

The stability of the binding between Bi1-G and PHA granules under different physicochemical conditions (temperature, ionic strength, and pH) was evaluated next. An aliquot of PHA granules containing Bi1-G was resuspended in buffer containing the specified condition and incubated for 2 h at the indicated temperature, or otherwise at 4°C, followed by separation of the supernatant and pellet fractions. As can be observed in Fig. 6, the protein mostly remained attached to the granule at the different temperatures (−20°C, 4°C, 37°C, and 60°C), ionic strengths (10 mM, 100 mM, and 1 M NaCl), and pHs (pH 3.0, pH 5.0, pH 7.0, and pH 9.0) tested, although some degradation occurred upon incubation at pH 3.0.

### Influence of fusion proteins on PHA production and functionalization efficiency.

The Bi1 segment was considered a promising choice as an optimized PHA affinity tag due to the small size of the fragment, colocalization with PHA granules *in vivo*, and its persistent interaction with PHA *ex vivo* under different physicochemical conditions, comparable to the BioF-PHA interaction. With the aim of studying the potential of Bi1 to be used as an affinity tag, the effect of the production of this protein on PHA production and PHA granule functionalization was evaluated. Strains producing BioF-G and Bi1-G, or harboring an empty vector as a control, were analyzed for PHA production (Table 1). The presence of any plasmid, whether empty vector or containing BioF-G- or Bi1-G-encoding genes, resulted in a roughly 20% decrease in PHA production in recombinant strains. Interestingly, in the *Δpha+C1* background, the presence of the whole BioF increased the percentage of PHA compared to that of Bi1-G-producing or empty vector cells, 38% versus ∼27%, respectively. Conversely, this effect was not maintained in the wild-type strain, where the PHA accumulation was similar among all the strains containing plasmid. The concentration of granule-associated proteins was also measured by quantifying bands from SDS-PAGE-isolated PHA granules. In the Δ*pha+C1* background, a larger amount of granule-associated BioF-G (8 mg per gram of PHA) was observed compared to that of Bi1-G (5.8 mg per gram of PHA). However, in molar terms, little difference was detected between the two segments, with the presence of protein at 0.17 μM and 0.16 μM per g of PHA for BioF-G and Bi1-G, respectively.

**TABLE 1:** Quantification of PHA production by GC-MS and determination of PHA granule-associated protein

Strain^*a*^	CDW^*b*^ (g/liter)	PHA (% of CDW)	Protein on granule surface (mg/g PHA)	Protein on granule surface (μM/g PHA)
KT2440 (WT)	1.47 ± 0.04	70 ± 4		
KT2440(pSP1)	0.79 ± 0.00	51 ± 2		
KT2440(pSP1BioF-G)	0.90 ± 0.03	54.7 ± 0.6	6 ± 1	0.14 ± 0.03
KT2440(pSP1Bi1-G)	0.98 ± 0.05	54.7 ± 2.4	7.4 ± 1.3	0.2 ± 0.04
KT2440(pSP1Bi2-G)	0.82 ± 0.01	55 ± 2		
*Δpha+C1*	0.73 ± 0.04	23 ± 2		
*Δpha+C1*(pSP1)	0.63 ± 0.00	28 ± 4		
*Δpha+C1*(pSP1BioF-G)	0.74 ± 0.05	38 ± 3	8.0 ± 0.7	0.17 ± 0.01
Δ*pha+C1*(pSP1Bi1-G)	0.65 ± 0.01	27 ± 4	5.8 ± 0.9	0.16 ± 0.02
*Δpha+C1*(pSP1Bi2-G)	0.59 ± 0.01	27 ± 3		

a WT, wild type.

b CDW, cell dry weight.

### Construction of a plasmid based on Bi1 for PHA functionalization.

**FIG 7:**
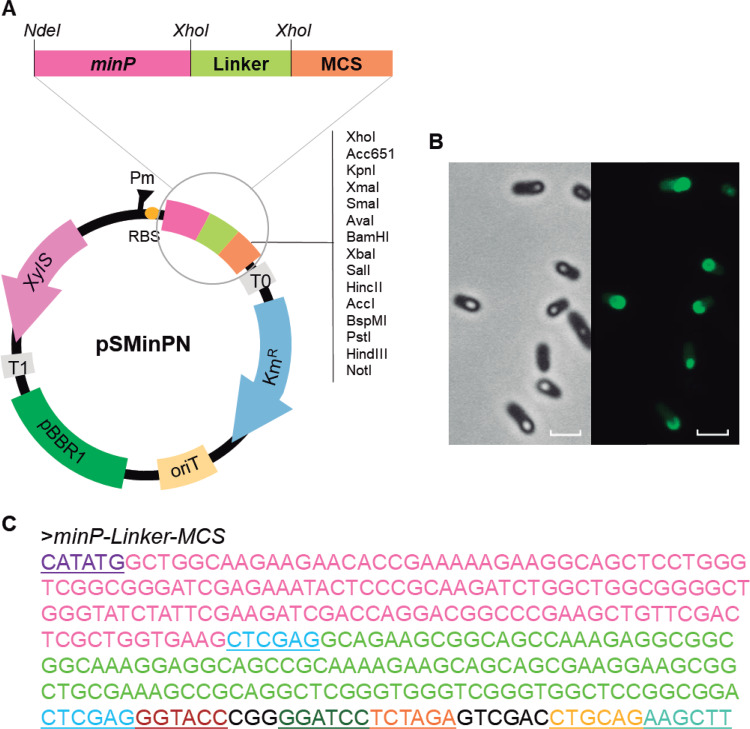
(A) pSMinPN plasmid derived from pSEVA238 to generate MinP N-terminal fusion proteins for the production of customized protein functionalized PHA nanobeads. (B) *In vivo* localization of the MinP-GFP produced by P. putida KT2440 *Δpha+C1* (pSMinP-1) after 24 h of growth under PHA-producing conditions. Scale bars, 2 μm. (C) Nucleotide sequence of the *minP*-linker-MCS construction. The *minP* gene is represented in pink and linker region in light green, and restriction sites are underlined with different colors (NdeI, purple; XhoI, dark blue; KpnI, red; BamHI, green; PstI, orange; XhoI, yellow; HindIII, light blue).

The Bi1 fragment demonstrated an interaction with PHA granules with superior functionalization efficiency and strong stability, making this construct suitable to be used as a tag to display functional proteins on the surface of PHA materials. Based on this optimized tag, here named MinP, a fusion plasmid based on pSEVA238 was designed to simplify the production of protein-functionalized PHA nanobeads. The MinP fusion plasmid allows for induced expression under the control of the *xylS*/P*m* system and was modified to incorporate a ribosome binding site followed by the MinP tag to allow N-terminal fusions of the protein of interest (Fig. 7A). The novel plasmid, called pSMinPN, contains a multicloning site (MCS) separated from the *minP* sequence by a glycine-rich linker region (Linker), which can be removed, if desired, by using two engineered flanking XhoI sites. This linker can also be replaced by other spacer regions or can include an intein site, expanding its potential applications. As a proof of concept, a *minP-gfp* fusion gene was constructed in pSMinPN and transformed into the *Δpha+C1* strain, and colocalization of MinP-GFP with PHA granules was observed by fluorescence microscopy following induction (Fig. 7B).

### Validation of the MinP tag for enzyme immobilization.

To validate MinP as an affinity tag, two enzymes, the β-galactosidase and the CueO oxidase from Escherichia coli, were genetically fused to *minP* by using the designed plasmid pSMinPN. Both enzymes are of wide applicability in the food industry for manufacturing lactose-hydrolyzed products and the production of galactosylated products, in the case of the β-galactosidase (35), and for the textile and paper industries as well as for organic compound degradation with CueO oxidase (15, 36). The β-galactosidase-coding gene was introduced by conventional cloning into XhoI and BamHI sites, resulting in the pSMinP-2 plasmid, and the *cueO* gene into XhoI and HindIII sites, resulting in the plasmid pSMinP-3. Both were independently transferred to the P. putida KT2440 Δ*pha+C1* strain, and both KT2440 Δ*pha+C1*(pSMinP-2) and KT2440 Δ*pha+C1*(pSMinP-3) strains were able to produce the fusion proteins of 122 kDa and 59 kDa, respectively (Fig. S2). The fusion proteins were able to bind to the PHA granules, and the binding stability under different physicochemical conditions (temperature, ionic strength, and pH) was evaluated as described above. As can be observed in Table 2, the fusion proteins remained attached to the granules at percentages of ≥85% under all conditions for both MinP–β-galactosidase and MinP-CueO (Table 2), confirming the suitability of the MinP system for immobilizing recombinant proteins into PHA granules. Maximal release was observed for MinP-CueO at pH 9.0 (17%) and for MinP–β-galactosidase at 37°C (24%). The latter was degraded after the treatment at 60°C.

**TABLE 2:** Percentage of granule-attached protein after 2 h of the corresponding treatment

Parameter	Mean % protein after treatment with:	
	MinP-CueO	MinP–β-galactosidase
pH		
3	97 (±2)	94 (±10)
5	96 (±3)	94 (±10)
7	94 (±5)	97 (±1)
9	83 (±13)	89 (±8)
[NaCl], mM		
0	86 (±8)	89 (±3)
10	96 (±2)	91 (±2)
100	87 (±8)	98 (±1)
1,000	91 (±8)	96 (±6)
Temperature, °C		
−20	92 (±7)	85 (±9)
4	96 (±4)	84 (±9)
37	91 (±5)	76 (±3)
60	91 (±5)	ND^*a*^

a ND, not detected.

Additionally, the release of the fusion proteins was tested in the presence of different percentages of Triton X-100 (Fig. S3). A pattern similar to that observed for Bi1-G was obtained for the MinP–β-galactosidase, where 0.15% (vol/vol) detergent treatment yielded higher release of protein from the granules than the assay carried out at 1.5% (vol/vol) Triton X-100. However, in the case of MinP-CueO, both detergent percentages resulted in similar protein release. These results suggest that the release of MinP fusions is not proportional to the detergent concentration and, together with those depicted in Fig. 5, indicate that the elution yields strongly depend on the fused polypeptide and do not follow a simple trend with detergent concentration. This behavior variety also has been found with the interaction of different BioF fusions with polyhydroxybutyrate granules (14) and suggests that detergent elution is a complex phenomenon involving both the affinity tag and the fused moiety, which deserves further molecular investigation.

**FIG 8:**
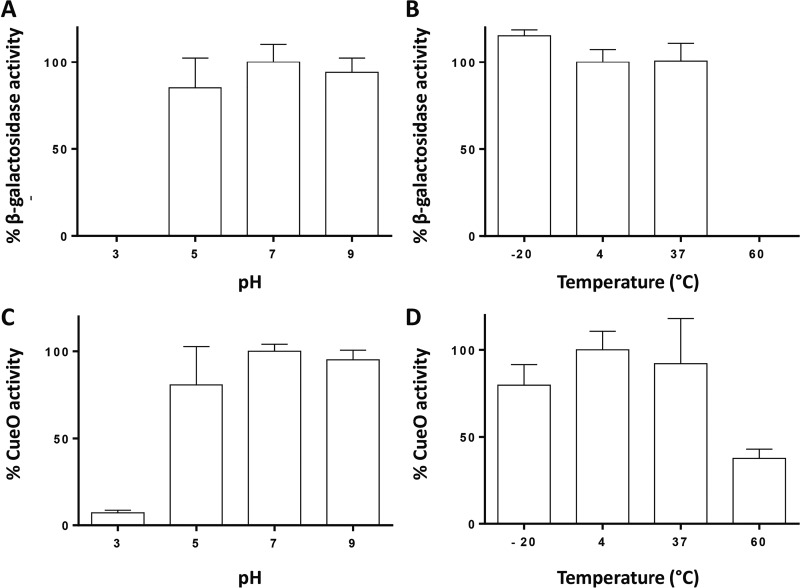
Percentage of activity retained by the protein immobilized at the granule after treatment at different pHs, with respect to a control at pH 7.0 (left), or different temperatures, with respect to a control at 4°C (right), for both MinP–β-galactosidase (A and B) and MinP-CueO (C and D). Error bars represent SD from 3 to 5 biological replicates.

To further validate the MinP tag system, the activity of each fusion enzyme was measured after treatment at different pHs (3.0, 5.0, 7.0, and 9.0) and temperatures (–20°C, 4°C, 37°C, and 60°C). As can be observed in Fig. 8, immobilized MinP–β-galactosidase preserves the enzyme activity, except at pH 3.0 (Fig. 8A) and 60°C (Fig. 8B). Similar results were obtained with immobilized MinP-CueO (Fig. 8C and D), suggesting that either the proteins, the polymer support, or both are affected under these extreme conditions.

Finally, the functionalization ratio was calculated as explained above for both enzymes. The ratios of milligram of protein per gram of PHA were 16.8 ± 1.6 for MinP–β-galactosidase and 8.5 ± 0.4 for MinP-CueO. However, when calculated in molar terms, both proteins had similar functionalization ratios of 0.14 ± 0.03 μM per g of PHA for MinP–β-galactosidase and 0.14 ± 0.06 μM per g of PHA for MinP-CueO, consistent with the results for MinP-GFP (0.16 μM per gram of PHA).

## DISCUSSION

This work addressed two main goals: to gain a better understanding of the binding capacity of BioF to the PHA granule and to use this knowledge to obtain improved versions of the tag that expand its biotechnological potential.

Many GAPs have been exploited as tags for the functionalization of PHA, especially PHA synthases and phasins. The mechanisms of the binding of these tags to the PHA material and the strength of the interaction are different, as are the properties of each tag, making them suitable depending on the target application. Phasins constitute an attractive tool for tuning PHA materials due to their strong affinity to PHA. Despite only a few studies having addressed the structural aspects of phasins, it is known that some common characteristics include the high proportion of disordered regions (28, 37), the presence of residues in α-helical conformation that increases in the presence of PHA (9, 28), and their tendency to form oligomers in solution (8, 28, 29, 38).

The PhaF phasin from P. putida constitutes an attractive biotechnology tool, as its N-terminal domain (the BioF affinity tag) binds to PHA granules by nonspecific hydrophobic interactions. In this work, various polypeptides based on BioF have been designed, differing in polarity, amphipathicity, and length: Bi1, Bi2, Bi3, and Bi4 (Fig. 2). All of them, except Bi2, were able to bind *in vivo* to PHA granules, suggesting that substrate recognition does not reside in a specific region (Fig. 3). Bi2 was unable to bind to the granule, likely due to its short size preventing the establishment of sufficient hydrophobic interactions with the polymer. The binding of the BioF-based peptides to PHA granules was demonstrated to be independent of the presence of other GAPs, such as phasins and depolymerase, on the surface of the granule (Fig. 4). The presence of PhaC1 and FadD could not be avoided due to their essential function for PHA accumulation, but our results indicate that the presence of most of the GAPs is dispensable for BioF segment binding.

The affinity of the truncated forms of the BioF tag to PHA was tested using various approaches. Bi3 displays a PHA localization pattern similar to that of BioF (Fig. 3B), although with a lower binding strength (Fig. 5). On the other hand, and although localized predominantly on the PHA granule surface by microscopy (Fig. 3A), the Bi4 segment did not show effective binding to isolated PHA granules (Fig. 3B). Previous studies have demonstrated that the inclusion of a functional leucine zipper motif is important for the oligomerization of PhaF (8, 28, 29). However, as both Bi3 and Bi4 share the same leucine zipper region, these effects put into question the crucial relevance of this motif for PHA affinity. The Bi1 fragment, containing the BioF N-terminal region, exhibited a PHA binding affinity similar to that of whole BioF, whereas Bi3, containing the C-terminal region of BioF, displayed reduced binding strength, despite both peptides sharing a common region that corresponds to the Bi2 segment. This divergence could be attributed to the different hydrophobic and amphipathic characteristics of both polypeptides (Fig. 1) rather than solely being a function of their lengths. In fact, mean hydrophobicity seems to be a dominant factor for PHA binding more so than amphipathicity, as the Bi1 fragment displays a clear higher value of <*H*> than Bi3, while it presents only a moderate value for the hydrophobic moment <μ*H*> (Fig. 2A). Moreover, Bi3 also contains a region that is presumably natively unfolded in solution that may result in an increased propensity for proteolytic attack (28). The two Bi3 bands observed in Fig. 3B may be due to this feature.

The 48-amino-acid segment Bi1 was chosen as an optimized tag for PHA functionalization and was renamed MinP. Its binding capacity under different physicochemical conditions was comparable or even stronger than that of full-length BioF (Fig. 3 to 5) (6, 14, 29). The stability of the binding between the tag and PHA is important to apply these devices across a variety of physicochemical conditions, as many industrial processes take place at extreme temperatures or pH (39, 40), conditions under which we demonstrate that MinP is retained at the surface of the PHA granule (Fig. 5 and 6 and Table 2). The high stability of MinP on the surface of the PHA granule constitutes a good choice for a strong immobilization of proteins and complements the diversity of PHA affinity tags tailored to any need.

The amount of fusion protein attached to PHA granules was observed to be similar for BioF and MinP in both wild-type and Δ*pha+C1* strains (Table 1). Likewise, similar functionalization in molar terms was obtained for MinP-CueO and MinP–β-galactosidase fusions. This suggests that the PHA-binding capability of BioF mainly resides in its N-terminal moiety and that the presence of other GAPs on the surface of the granule does not affect the functionalization efficiency when the levels of the recombinant protein inside the cell are much higher than those of other GAPs, as was previously observed (30).

Phasins have a positive effect on PHA production and PHA granule biogenesis (27, 31). This has been proposed to be related to the interfacial role of phasins to segregate the hydrophobic PHA granules away from hydrophilic cytoplasm, avoiding potential deleterious effects of PHA production (41, 42). We observed the positive effects of the production of phasin segments in Table 1, where the presence of BioF increased the amount of PHA produced in the Δ*pha+C1* strain, while MinP production did not. BioF tag presence likely favors granule formation and, thus, improves PHA production, whereas MinP appears not to have complete phasin activity. This could be due to the lack of the leucine zipper motif in MinP, which is important for oligomerization and to establish the layer of GAPs that covers the PHA granule (29). The segregation of PHA granules, driven by the natural PhaF (27), is affected in the absence of this protein, very likely influencing PHA accumulation. This decrease in production could be solved by introducing the natural phasin PhaF if an industrial exploitation of the system that requires maximization of the PHA yield would be envisaged.

Finally, we propose a construction based on plasmid pSEVA238 to implement the production of fusion proteins at the C-terminal domain of the MinP tag and containing a movable linker as well as an MCS for cloning. The SEVA plasmids constitute a versatile platform for swapping modules as required (http://seva.cnb.csic.es/). Thus, the MinP module can be incorporated into the standardized synthetic biology library as a novel brick for polyester functionalization.

## MATERIALS AND METHODS

### Strains, media, and growth conditions.

The bacterial strains used in this study are listed in Table 3. E. coli and P. putida strains were grown routinely in lysogeny broth (LB) medium (43) at 37°C and 30°C, respectively, in a horizontal shaker at 200 rpm. Solid media were supplemented with 1.5% (wt/vol) agar. Kanamycin antibiotic was added when needed at a final concentration of 50 μg/ml.

**TABLE 3:** Strains, plasmids, and oligonucleotides used in this work

Strain	Description^*a*^	Reference or source
Pseudomonas putida		
KT2440	Wild-type strain derived from P. putida mt-2 cured of the pWW0 plasmid	51
KT2440 *Δpha*	Strain with the *pha* cluster deleted	CECT 30020
KT2440 *Δpha*+*C1*	Strain with *phaC1* genes integrated into the genome of KT2440 *Δpha* under the control of *P_14a_* promoter	This work
Escherichia coli		
DH10B	Host for E. coli plasmids	Invitrogen, Thermo Fisher Scientific, USA
HB101	Host for plasmid pRK600	52
CC118λpir	Host strain for plasmid pTnS-1	53
DH5αλpir	Km^r^, XylS/*Pm*, pBBR1 ori	Prieto laboratory collection
Plasmids		
pSEVA238	Km^r^, XylS/*Pm*, pBBR1 ori	34
pSEVA225	Km^r^, LacZ, RK2 ori (source of β-galactosidase)	34
pCueO	pASK-IBA3 derivative containing the *cueO* sequence; Cm^r^, f1 ori	50
pSP1	pSEVA238 derivate containing an RBS cloned into XbaI and HindIII sites	This work
pSP1Bi1-G	pSP1 derivate harboring the fusion gene *bi1-gfp*	This work
pSP1Bi2-G	pSP1 derivate harboring the fusion gene *bi2-gfp*	This work
pSP1Bi3-G	pSP1 derivate harboring the fusion gene *bi3-gfp*	This work
pSP1BioF-G	pSP1 derivative harboring the fusion gene *bioF-gfp*	This work
pSP1Bi4-G	pSP1 derivate harboring the fusion gene *bi4-gfp*	This work
pSMinPN	pSP1 derivate containing the *minP* sequence followed by a glycine-rich region and an MCS	This work
pSMinP-1	pSMinPN derivative containing the GFP sequence cloned into XhoI and HindIII sites	This work
pSMinP-2	pSMinPN derivative containing the *lacZ* sequence cloned into XhoI and BamHI sites	This work
pSMinP-3	pSMinPN derivative containing the *cueO* sequence cloned into XhoI and HindIII sites	This work
pTn7	Km^r^, Gm^r^, ori R6K, Tn*7*L and Tn*7*R extremes, standard multiple-cloning site	45
pTn7-ModC1	pTn*7* derivate harboring *phaC1* gene	This work
pMAB20-GFP-LYTAG	pMAB20 derivate harboring the fusion gene *bioF-gfp-lytag*	54
pRK600	Cm^r^, ColE1 *oriV RK2* Mob^+^ Tra^+^ donor of transfer functions	55
pTnS-1	Ap^r^, ori R6K, *TnSABC+D* operon	56
Oligonucleotide primers		
GFP-F	GGAAGTCCATATGGAACCGCTCGAGATGATCATGGGAATTCATAA	
GFP-R	CATGCAAAGCTTTATTTGTAGAGTTCATCCATGCCG	
Bio1-F	GGAAGTCCATATGGCTGGCAAGAAGAACAC	
Bio1-R	ATGCACTCGAGCTTCACCAGCGAGTCGAACA	
Bio3-F	GGAAGTCCATATGCTTAACAGCGCCATCTCG	
Bio3-R	ATGCACTCGAGGCGCGACGAAATCGGCGTAA	
Bio4R	ATGCACTCGAGAGTGGTCGAAGACTTGGCAGT	
MinPNt-F	GGAAGTCTGAATTCAATAATTTTGTTTAACTTTAAGAAGGAGATATACATATGGCTGGCAAGAAGAAC	
MinPNt-R	ATATCAAGCTTCTGCAGGTCGACTCT	
BamHI-GFP-F	GGAAGTCTGGATCCATGCGTAAAGGAGAAGAACT	
XhoI-GFP-R	ATATCCTCGAGTGGTACCGGCCACCCCCT	
XhoI-CueO-F	AGTCTTCTCGAGGCAGAACGCCCAACGTTACCGAT	
HindIII-CueO-R	TCAGAATTCGAAAATATGGCATTTGGGATTG	
XhoI-LacZ-F	TCTGGCTCGAGATGACCATGATTACGGATTCACTG	
BamHI-LacZ-R	AGTCTTGGATCCATTATTTTTGACACCAGACCAACTG	

a Underlined sequences in the oligonucleotides indicate restriction sites.

For PHA production, the experiments were performed in 0.1 N M63 [13.6 g KH_2_PO_4_, 0.2 g (NH_4_)_2_SO_4_, 0.5 mg FeSO_4_·7H_2_O to 1 liter, adjusted to pH 7.0 with KOH] containing 15 mM sodium octanoate. This is a nitrogen-limited medium for PHA production, where 0.1 N means a 10% nitrogen source concentration of the standard M63 medium (2 g liter^−1^). A 20-ml overnight culture grown in LB at 30°C and 200 rpm was washed with 10 ml 145 mM NaCl, inoculated at an OD_600_ of 0.3 in 100 ml 0.1 N M63 medium, and grown at 30°C, as previously described (30). Minimal medium was supplemented with 1 mM MgSO_4_ and 0.1 ml of a solution of trace elements (for 1 liter, 2.78 g FeSO_4_·7H_2_O, 1.98 g MnCl_2_·4H_2_O, 2.81 g CoSO_4_·7H_2_O, 1.47 g CaCl_2_·2H_2_O, 0.17 g CuCl_2_·2H_2_O, 0.29 g ZnSO_4_·7H_2_O in 1 N HCl). When necessary, 3-methylbenzoate (3-MB) was added at a final concentration of 1 mM for the induction of pSEVA238 derivative plasmids (34).

### DNA manipulation, plasmid, and strain constructions.

DNA manipulations were performed as previously described (43), and the whole oligonucleotides used are listed in Table 3.

**FIG 9:**
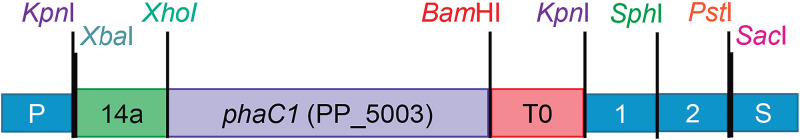
*pha* minimal cluster for the construction of P. putida KT2440 Δ*pha+C1*. 14a, promoter (45); T0, lambda T0 terminator; *phaC1*, PhaC1 synthase coding gene from P. putida KT2440; P, S, 1, and 2, overlap regions for Gibson Assembly technique (44).

For the construction of the strain carrying the PhaC1 polymerase, a modular cluster was *in silico* designed (Fig. 9). The synthesis was carried out by GenBank. The cluster allows the introduction of other modules through the Gibson Assembly technique (44) by using the overlap neutral regions (named 1 and 2). In addition, promoters and cargo genes can be easily exchanged using XbaI/XhoI/BamHI restriction sites. The cluster is flanked by two NotI sites to be cloned into the pTn*7*-M transposon for integration in the P. putida KT2440 *Δpha* genome. The *phaC1* gene was introduced under the control of a constitutive synthetic promoter (*P_14a_* promoter) (45), and the corresponding strain was called the P. putida KT2440 Δ*pha+C1* strain. The *M1-PhaC1-T0* module was synthetized by modifying the nucleotide sequence (see Fig. S4 in the supplemental material) to avoid the restriction sites specified in Fig. 9 and introduced in the pTn*7*-M plasmid for integration in the P. putida KT2440 Δ*pha* genome (Table 3).

For the construction of the pSMinPN plasmid, the sequence containing the *minP* gene followed by a linker and flanked by XhoI restriction sites and a multiple cloning site (MCS) was designed and synthesized. The construct was amplified using primers minPNt-F and minPNt-R (Table 3) and cloned into pSEVA238 by using the EcoRI and HindIII restriction enzymes. The gene coding for green fluorescent protein (GFP) was cloned into the pSMinPN plasmid between the XhoI and HindIII sites to generate pSMinP-1 and transformed by electroporation into the P. putida KT2440 Δ*pha+C1* strain (Table 3). For the construction of the pSMinP-2 and pSMinP-3 plasmids, the genes coding for β-galactosidase (*lacZ*) and CueO (*cueO*) were amplified from pSEVA225 and pCueO, respectively (Table 3), using the XhoI-LacZ-F and BamHI-LacZ-R oligonucleotides for *lacZ* and XhoI-CueO-F and HindIII-CueO-R for *cueO* (Table 3). Each one was cloned onto the 3′ end of the MinP tag of pSMinPN plasmid between the XhoI/BamHI and XhoI/HindIII sites for *lacZ* and *cueO*, respectively. Plasmids pSMinP-2 and pSMinP-3 were independently transferred by electroporation to the P. putida KT2440 Δ*pha+C1* strain.

### Protein structure prediction.

All predicted α-helical sequences (18-aa windows) were analyzed for their hydrophobicity and amphipathicity with the HeliQuest utilities (http://heliquest.ipmc.cnrs.fr/) (46), using the Fauchere and Pliska scale (47) to calculate the mean hydrophobicity (<*H*>), while the mean hydrophobic moment (<μ*H*>) was calculated according to Eisenberg et al. (48). The hydrophobic moment is a measure of the amphipathicity of a helix where each amino acid is assigned a value (positive or negative) according to its hydrophobicity. Mean hydrophobicity is the sum of the hydrophobicity values divided by the number of residues (GenScript).

### BioF library construction.

Based on structure prediction and the hydropathic profile of BioF, several potential PHA affinity tags were constructed (Fig. 2). Different PCR products were obtained using the oligonucleotides Bio1-F and Bio1-R (Bi1), Bio1-F and Bio4-R (BioF), Bio2-F and Bio1-R (Bi2), Bio3-F and Bio3-R (Bi4), and Bio2-F and Bio3-R (Bi3), with P. putida KT2440 genomic DNA as the template (Table 3). The corresponding DNA fragments were cloned into the pSP1 plasmid (Table 3) using NdeI and HindIII restriction enzymes, giving the plasmids pSP1Bi1, pSP1Bi2, pSP1Bi3, pSP1Bi4, and pSP1BioF (Table 2). To allow the *in vivo* localization of each BioF-derived polypeptide, the gene coding for the GFP was amplified from pMAB20-GFP-LYTAG plasmid (31) by using the GFP-F and GFP-R oligonucleotides (Table 3) and cloned onto the 3′ end of the Bi-G segments between the XhoI and HindIII restriction sites. Finally, the plasmids were transformed by electroporation into P. putida KT2440. The most promising designs were also introduced into the P. putida KT2440 *Δpha+C1* strain.

### Fluorescence microscopy.

P. putida KT2440 and P. putida KT2440 *Δpha+C1* strains carrying plasmid pSP1Bi1-G, pSP1Bi2-G, pSP1Bi3-G, pSP1Bi4-G, or pSP1BioF-G (Table 3) were grown in PHA, producing media for 24 h at 30°C and 200 rpm. The induction of the corresponding genes was carried out at an OD_600_ of 0.8 by the addition of 1 mM 3-MB. After 20 h of induction, the cells were directly observed through a Leica AF6000 LX wide-field multidimensional microscopy system equipped with a Hamamatsu C9100-02 charge-coupled device digital camera. Live cell imaging was performed using an 100×/1.4 oil immersion objective, and GFP fluorescence was collected using a GFP-3035C-000 Semrock filter, with excitation and emission ranges of 472/30 nm and 520/35 nm, respectively, and a 495-nm dichroic mirror.

### PHA granule isolation.

The PHA granules from P. putida KT2440 and P. putida KT2440 Δ*pha+C1* strains carrying the plasmid pSP1Bi1-G, pSP1Bi2-G, pSP1Bi3-G, or pSP1BioF-G were isolated by following the protocol previously described (31). Briefly, bacterial cells from 14 ml of culture at an OD_600_ of 2 to 3 (except where otherwise specified) were harvested by centrifugation at 12,000 × *g* for 20 min, suspended in 7 ml of 15 mM Tris-HCl, pH 8.0, and disrupted twice by French press (1,000 lb/in^2^). The resulting suspension was centrifuged for 30 min at 12,000 × *g*, and the pellet fraction was resuspended in 5 ml of 15 mM Tris-HCl, pH 8.0, and layered over 5 ml of 55% glycerol. The solution was centrifuged at 17,000 × *g* for 30 min, and the isolated granules were washed twice with 15 mM Tris-HCl, pH 8.0, to remove the residual glycerol and suspended into 0.5 to 1.5 ml of 15 mM Tris-HCl, pH 8.0.

### Stability of MinP proteins attached to the PHA granule.

An aliquot of 100 μl of the isolated granules from the KT2440 Δ*pha+C1* strain (see “PHA granule isolation,” above) containing the plasmid coding for the corresponding BioF-derived polypeptide were collected by centrifugation, suspended in Triton X-100 at different concentrations (0.015%, 0.15%, and 1.5%, vol/vol, in 15 mM Tris-HCl, pH 8.0), and incubated for 2 h at room temperature. The assay was later repeated with the final fusion proteins MinP-CueO and MinP–β-galactosidase.

The binding stability of Bi1-G protein, MinP-CueO, and MinP–β-galactosidase to the PHA granules was also tested under different temperature, pH, and ionic strength conditions as previously described (29). Briefly, an aliquot of 100 μl of purified granules from the P. putida KT2440 Δ*pha+C1* strain (pSP1Bi1-G, pSPMinP-2, and pSPMinP-3) (see “PHA granule isolation,” above) was incubated for 2 h under the following conditions: (i) in 100 μl of 15 mM Tris-HCl, pH 8.0, at −20, 4, 37, or 60°C; (ii) in 100 μl of 15 mM Tris-HCl, pH 8.0, with 0, 10, 100, or 1,000 mM NaCl at 4°C; or (iii) in 100 μl of 15 mM sodium citrate, pH 3.0 or 5.0, in 100 μl of 15 mM Tris-HCl, pH 7.0 or 9.0, at 4°C. In all cases after the 2-h incubation time, the granules were centrifuged at 12,000 × *g* for 15 min at 4°C, and the retained and soluble protein fractions were analyzed and estimated by SDS-PAGE as specified below. The percentage of discharged protein was obtained by subtracting the amount of protein liberated from the granules from the total amount of protein (sum of the soluble and insoluble fractions). Each assay was performed in duplicate.

### Protein quantification.

The content of the different BioF- and MinP-based tag proteins on the granule surface was determined by imaging the bands separated on 12.5% SDS-PAGE and estimating the amount of protein present using the software package ImageJ. Pellet and granule protein fractions of the crude extract and the granule fraction isolated from each culture were separated by SDS-PAGE and stained with Coomassie brilliant blue G-250 by following the described protocol (43). The amount of protein anchored to the surface of the granules was estimated by imaging the Coomassie-stained gels and taking into account the amount of isolated granules loaded in each well and calibrating against Precision Plus protein standards from Bio-Rad.

### PHA quantification.

Monomer composition and cellular content of PHA were determined by gas chromatography-mass spectrometry (GC-MS) by following the previously described protocol (49). Briefly, PHA monomers were obtained by acid methanolysis with 2 ml of methanol acidified with 15% (vol/vol) H_2_SO_4_ and 2 ml of 0.5 mg/ml 3-methyl benzoate in chloroform as an internal standard. This mixture was incubated for 5 h in an oil bath at 100°C. After cooling, 1 ml of distilled water was added to the mixture and centrifuged for 10 min at 3,000 × *g* to separate the phases. A second step of extraction was performed to remove the residual H_2_SO_4_. Finally, a small amount of H_2_SO_4_ powder was added to remove residual water. The organic layer was analyzed using an Agilent 7890A GC equipped with a DB-5HT capillary column (30-m length, 0.25-mm internal diameter, and 0.1-μm film thickness), and mass data were acquired and processed with an Agilent 5975C mass spectrometer. The oven temperature program was set at an initial temperature of 80°C for 2 min and then from 80°C up to 115°C at a rate of 5°C/min for the efficient separation of peaks. Spectra were obtained as electron impacts with an ionizing energy for MS operation of 70 eV.

Biomass calculation was carried out as previously described (49). Fifty milliliters of culture medium was centrifuged for 30 min at 3,000 × *g* at 4°C, and pellets were lyophilized for 24 h and weighed.

### Enzymatic activity assays.

Enzymatic activity was tested for the granule-immobilized fusion proteins after treatment at different pHs and temperatures, and relative activity was calculated regarding untreated granule-immobilized enzymes as follows.

**(i) β-Galactosidase assay.** First, to determine the specific activity (SA) of the immobilized enzyme, an aliquot of 12.5 μl of granules with a defined protein concentration (see “PHA granule isolation” and “Protein quantification,” above) was centrifuged (15 min, 16,060 × *g*, room temperature) and resuspended in 500 μl of 100 mM sodium phosphate, pH 7.0, in 2-ml tubes, to which 500 μl of buffer Z (60 mM Na_2_HPO_4_, 40 mM NaH_2_PO_4_, 10 mM KCl, 1 mM MgSO_4_, 50 mM β-mercaptoethanol, pH 7.0) was added. The tubes were prewarmed at 28°C for 3 min in a thermoblock (Fisher Scientific, USA), and subsequently 200 μl of *O*-nitrophenyl-β-galactopyranoside (ONPG) (Sigma-Aldrich, USA) (4 μg·ml^−1^) was added and incubated for 3 min more, after which the reaction was stopped by the addition of 500 μl 1 M Na_2_CO_3,_ pH 11.0. Previously, we had determined both the protein concentration and the reaction time needed to measure the activity in the linear part of the activity curve. The ONP generated in the stopped reaction was measured at 420 nm in a spectrophotometer (DU 520; Beckman) and used for the SA calculation (units per milligram of protein) by using an extinction coefficient of 4,800 M^−1 ^cm^−1^ for ONP. The SA of the immobilized MinP–β-galactosidase resulted in 6.8 × 10^4^ UA mg^−1^, in agreement with the SA reported for BioF-β-galactosidase (2.8 × 10^5^ UA mg^−1^) (30). A unit of activity, UA, is defined as the amount of protein needed to hydrolyze 1 nmol of ONPG per min at 28°C.

For the functional stability test, the granules containing the immobilized protein were previously treated as described in “Stability of MinP proteins attached to the PHA granule,” above (2 h at pH 3.0, 5.0, 7.0, or 9.0 and temperature of –20°C, 4°C, 37°C, or 60°C). The positive control was prepared with granules at pH 7.0 and at 4°C for pH and temperature treatment. As a negative control, granules extracted from the P. putida KT2440 *Δpha+C1* parental strain, incubated at 4°C for 2 h, were taken. The enzyme activity assay was undertaken as explained in the paragraph above, except that, after stopping the reaction, and for the ONP quantification, an aliquot of 200 μl of each suspension was transferred to a multiwell plate (Falcon, USA), and the absorbance at 420 nm was measured in a VersaMax microplate reader (Molecular Devices). Enzyme activity was calculated as Miller Units by using the following formula: 1,000 × (OD_420_)/(*T* × *P*), where *T* is the reaction time, in minutes, and *P* is the total amount of protein quantified by gel densitometry after the treatment (see “Protein quantification,” above). The enzyme activity in each case was shown as a percentage with regard to a control at pH 7.0 for the pH treatments and at 4°C for the temperature treatments.

**(ii) CueO assay.** To determine the SA of the immobilized enzyme, an aliquot of 50 μl of granules (see “PHA granule isolation,” above) with a defined protein concentration, as determined by gel densitometry, was centrifuged (15 min, 16,060 × *g* at room temperature) and resuspended in the same volume of 100 mM sodium phosphate, pH 6.5. The granule suspension was transferred to a spectrophotometer cuvette, and the reaction was triggered by the addition of CuCl_2_ (Fluka, Switzerland) and the CueO substrate, 2,6 dimethoxyphenol (DMP) (Sigma-Aldrich, USA), at final concentrations of 10 μM and 2 mM, respectively, in 100 mM sodium phosphate, pH 6.5, and in a final volume of 1 ml. The reaction product, 3,3′,5,5′-tetramethoxydiphenoquinone, was monitored at 30°C by measuring the absorbance at 468 nm (ε_468nm_ = 14,800 M^−1 ^cm^−1^) (50) every 5 min to ensure the activity in the linear part of the oxidation curve. The specific activity of the immobilized MinP-CueO was 2,300 UA mg^−1^, comparable with that of soluble CueO (6,000 UA mg^−1^), although slightly reduced, probably due to the presence of the MinP fused moiety. One UA is defined as the amount of enzyme needed to oxidize 1 nmol of DMP per min at 30°C.

For the functional stability test, the granules containing the immobilized protein were previously treated as described in “Stability of MinP proteins attached to the PHA granule,” above (2 h at pH 3.0, 5.0, 7.0, or 9.0 and temperature of –20°C, 4°C, 37°C, or 60°C). The positive control was prepared with granules at pH 7.0 and at 4°C for pH and temperature treatment, respectively. As a negative control, granules with heat-inactivated MinP-CueO (2 min, 98°C) were used. For relative quantification, the assays were carried out in a multiwell plate (Falcon, USA) in a final volume of 200 μl, and the activity was monitored in a multiwell reader at OD_468_ (VersaMax; Molecular Devices).

## Supplementary Material

Supplemental file 1
