# Supplementary material for: Dissecting the Polyhydroxyalkanoate-Binding Domain of the PhaF Phasin: Rational Design of a Minimized Affinity Tag
Source: Appl Environ Microbiol. 2020 Jun 2;86(12):e00570-20. doi: 10.1128/AEM.00570-20 (PMC7267194; doi:10.1128/AEM.00570-20)
Supplement: Supplemental file 1 [file AEM.00570-20-s0001.pdf]

Supplemental material

DISSECTING THE POLYHYDROXYALKANOATE-BINDING DOMAINS OF THE PhaF PHASIN: RATIONAL DESIGN OF A MINIMIZED AFFINITY TAG

Running title: Rational design of a minimized PHA affinity tag

Aranzazu Mato [a,b, †], Francisco G. Blanco [a,b, †], Beatriz Maestro[c], Jesús M. Sanz[c,d], Jesús Pérez-Gil[e] and M. Auxiliadora Prieto[a,b*]

[a]Polymer Biotechnology Group. Microbial and Plant Biotechnology Department, Centro de Investigaciones Biológicas Margarita Salas-CSIC, Madrid, Spain.

[b]Interdisciplinary Platform for Sustainable Plastics towards a Circular Economy-Spanish National Research Council (SusPlast-CSIC), Spain.

[c]Host-parasite Interplay In Pneumococcal Infection Group. Microbial and Plant Biotechnology Department, Centro de Investigaciones Biológicas Margarita Salas-CSIC, Madrid, Spain.

[d]Centro de Investigación Biomédica en Red de Enfermedades Respiratorias (CIBERES), Madrid, Spain

[e]Biochemical and Molecular Biology Department, Facultad de Ciencias Biológicas, Universidad Complutense de Madrid, Madrid, Spain.

[†]These authors contributed equally to this work. Order was decided by seniority.

* Corresponding Author. mailto:auxi@cib.csic.es

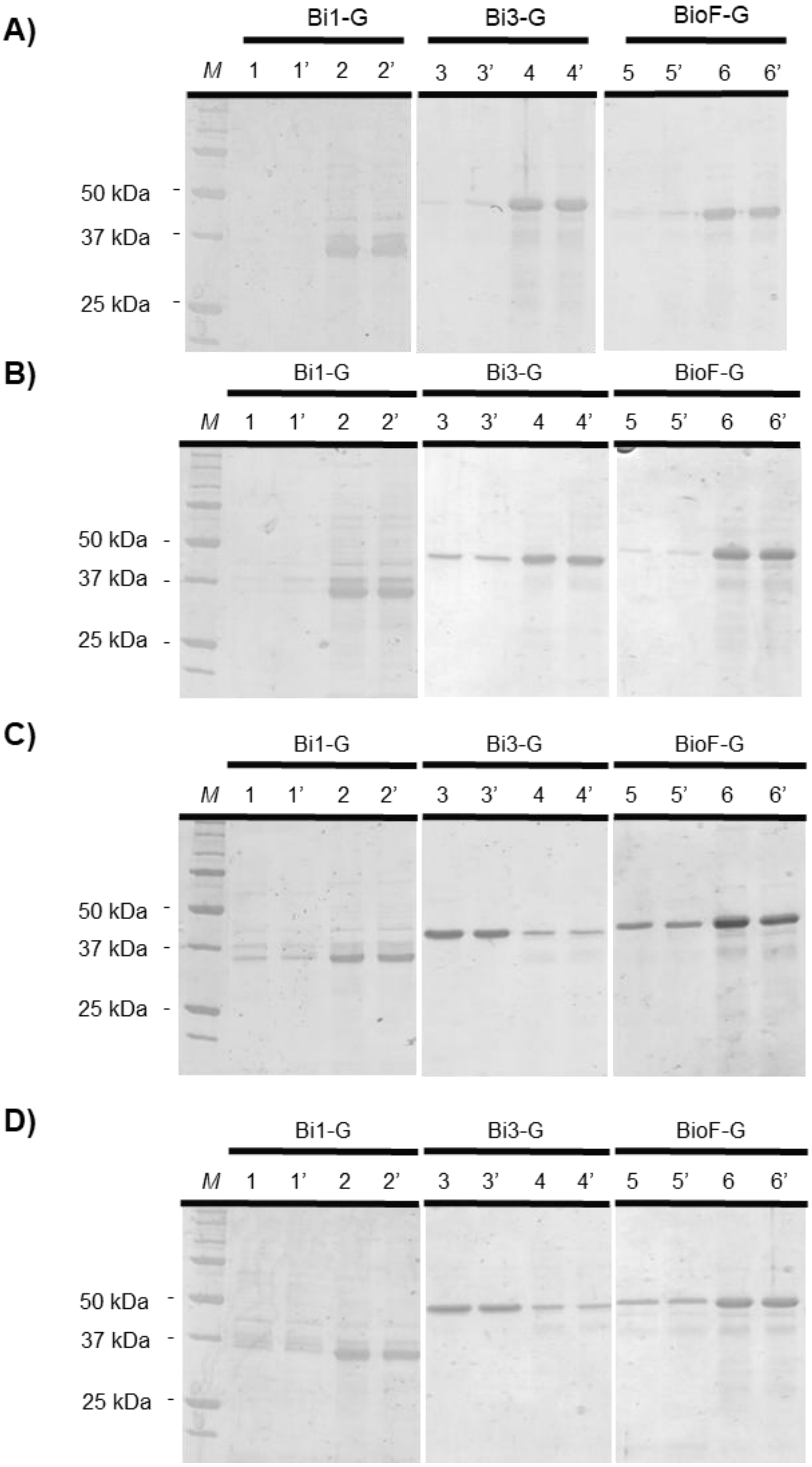
A)
Bi1-G
Bi3-G
BioF-G
M 1 1' 2 2' 3 3' 4 4' 5 5' 6 6'
50 kDa
37 kDa
25 kDa
B)
Bi1-G
Bi3-G
BioF-G
M 1 1' 2 2' 3 3' 4 4' 5 5' 6 6'
50 kDa
37 kDa
25 kDa
C)
Bi1-G
Bi3-G
BioF-G
M 1 1' 2 2' 3 3' 4 4' 5 5' 6 6'
50 kDa
37 kDa
25 kDa
D)
Bi1-G
Bi3-G
BioF-G
M 1 1' 2 2' 3 3' 4 4' 5 5' 6 6'
50 kDa
37 kDa
25 kDa

Figure S1. Coomassie stained SDS-PAGE of PHA granules extracted from *P. putida* KT2440 *Δpha+C1* expressing Bi1-G, Bi3-G or BioF-G. A) The stability of the Bi-G segments interacting with PHA granules was assessed after 2 h at room temperature in the absence of Triton. Lane M, molecular weight marker; Lanes 1, 1’, 3, 3’, 5 and 5’ respective Bi segments replicates in soluble fraction after 2 h at room temperature; Lanes 2, 2’, 4, 4’, 6 and 6’ pellet fraction replicates of respective Bi segments retained on PHA granule after 2 h at room temperature. B) The stability of the Bi-G segments interacting with PHA granules was assessed by exposure to 0.015 % Triton X- 100 for 2 h. Lane *M*, molecular weight marker; Lanes 1, 1’, 3, 3’, 5 and 5’, respective Bi segments replicates in soluble fraction after 2 h in 0.015 % Triton X-100; Lanes 2, 2’, 4, 4’, 6 and 6’, pellet fraction replicates of respective Bi segments retained on PHA granule after 2 h in 0.015 % Triton X-100 C) The stability of the Bi-G segments interacting with PHA granules was assessed by exposure to 0.15 % Triton X- 100 for 2 h. Lane *M*, molecular weight marker; Lanes 1, 1’, 3, 3’, 5 and 5’, respective Bi segments replicates in soluble fraction after 2 h in 0.15 % Triton X-100; Lanes 2, 2’, 4, 4’, 6 and 6’, pellet fraction replicates of respective Bi segments retained on PHA granule after 2 h in 0.15 % Triton X-100. D) The stability of the Bi-G segments interacting with PHA granules was assessed by exposure to 1.5 % Triton X- 100 for 2 h. Lane *M*, molecular weight marker; Lanes 1, 1’, 3, 3’, 5 and 5’, respective Bi segments replicates in soluble fraction after 2 h in 1.5 % Triton X-100; Lanes 2, 2’, 4, 4’, 6 and 6’, pellet fraction replicates of respective Bi segments retained on PHA granule after 2 h in 1.5 % Triton X-100 Volumes loaded correspond to 15 μL of the supernatant and retained fractions obtained from the initial quantity of granules.

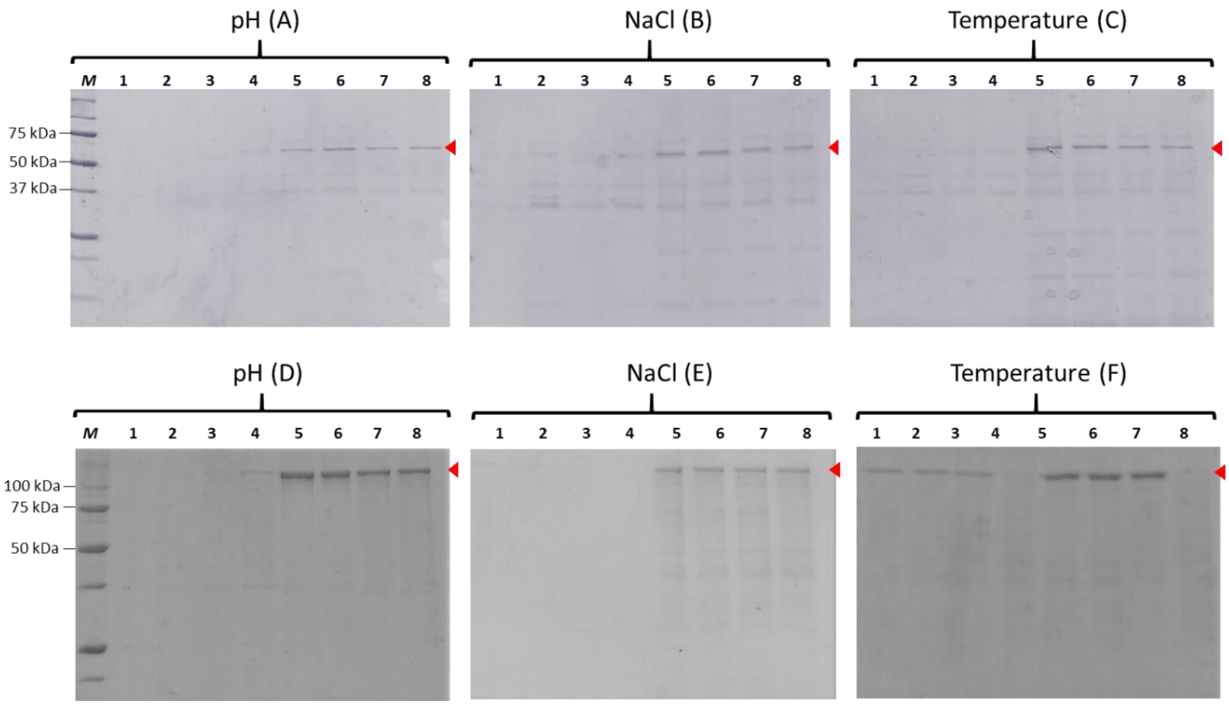

Fig S2. Coomassie stained SDS-PAGE of PHA granules extracted from *P. putida* KT2440 *Δpha+C1* expressing MinP-CueO of MW 59 kDa (upper panels) and MinP-β-galactosidase of MW 122 kDa (lower panels). The stability of fusion proteins interacting with PHA granules was assessed by their exposure to a range of pH (A, D), ionic strength (B, E) and temperatures (C, F) for 2 h. A, D) Lane *M*, molecular weight marker. Lanes 1- 4, released soluble fraction after treatment with pH 3.0, 5.0, 7.0 and 9.0, respectively. Lanes 5- 8, PHA granule retained protein fraction after treatment at pH 3.0, 5.0, 7.0 and 9.0, respectively. B, E) Lanes 1-4, released soluble fraction after treatment with 0, 10, 100 and 1000 mM NaCl, respectively. Lanes 5- 8, PHA granule retained protein fraction after treatment with 0, 10, 100 and 1000 mM NaCl, respectively. C, F) Lanes 1- 4, released soluble fraction after at -20 °C, 4 °C, 37 °C and 60 °C, respectively. Lanes 5-8, PHA granule retained protein fraction after treatment at -20 °C, 4 °C, 37 °C and 60 °C, respectively. Volumes loaded correspond to 12.5 μL of the soluble and

insoluble fractions obtained after the treatment of the granules. Red arrows indicate the predicted molecular weights of the recombinant proteins.

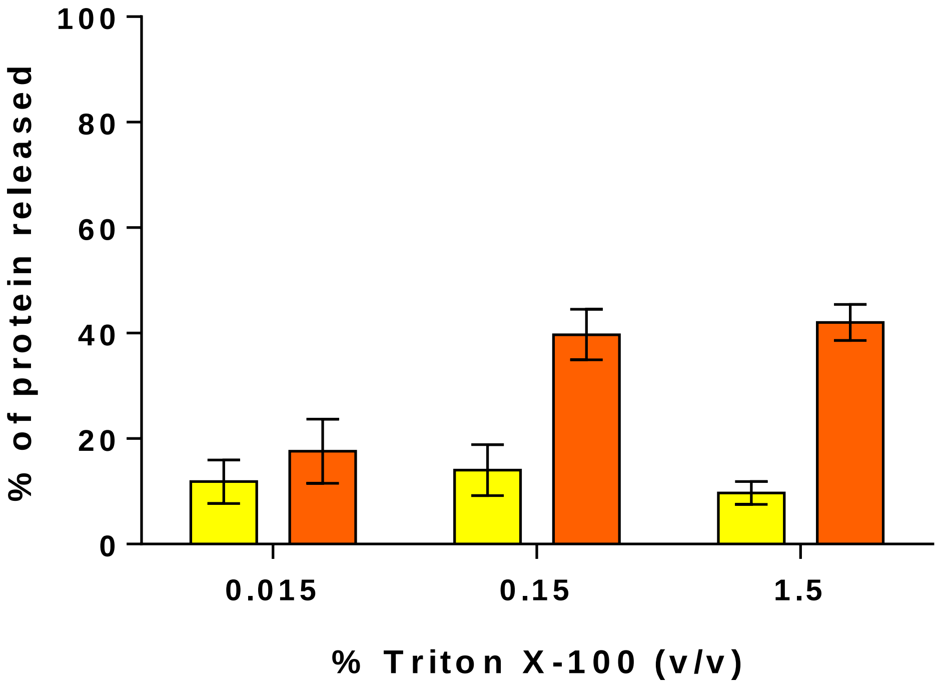

Fig S3. Percentage of protein release from PHA granules after 2 h of incubation at room temperature with 0.015 %, 0.15 % or 1.5 % (v/v) Triton X-100 for different MinP-fusion proteins. Yellow bars represent MinP-β-galactosidase release and orange bars represent MinP-CueO release. Error bars represent SD from three biological replicates.

>PhaC1_mod

ATGAGTAACAAGAACAACGATGAGCTACAGCGGCAGGCCTCGGAAAACACCCTGGGGCTGAACCC

GGTCATCGGCATCCGCCGCAAGGACCTGTTGAGCAGCGCACGCACCGTGCTGCGCCAGGCCGTGC

GCCAACCGCTGCACAGCGCCAAGCATGTGGCTCACTTTGGCCTGGAGCTGAAGAACGTGTTGCTG

GGCAAATCCAGCCTGGCCCCGGACAGCGACGACCGTCGCTTCAATGACCCGGCCTGGAGCAACAA

CCCGCTGTACCGCCGCTACCTGCAAACCTACCTGGCCTGGCGCAAGGAGCTGCAAGACTGGGTGA

GCAGCAGCGACCTGTCCCCCCAGGACATCAGCCGCGGCCAGTTCGTCATCAACCTGATGACCGAG

GCCATGGCGCCGACCAATACCCTGTCCAACCCGGCTGCGGTCAAACGCTTCTTCGAAACCGGCGG

CAAGAGCCTGCTCGATGGCCTGTCCAACCTGGCCAAGGACATGGTCAACAACGGCGGTATGCCCA

GCCAGGTGAACATGGATGCCTTCGAAGTGGGCAAGAACCTGGGCACCAGCGAAGGCGCGGTGGTG

TACCGCAACGATGTGCTGGAACTGATCCAGTACAGCCCCATCACCGAGCAGGTGCACGCCCGTCC

GCTGCTGGTGGTGCCACCGCAGATCAACAAGTTCTACGTGTTCGACCTCAGCCCGGAAAAGAGCC

TGGCGCGCTTCTGCCTGCGCTCGCAGCAGCAGACCTTCATCATCAGCTGGCGCAACCCGACCAAG

GCCCAGCGTGAATGGGGCCTGTCCACCTACATCGATGCGCTGAAAGAAGCCGTCGACGCGGTGCT

GTCGATTACCGGCAGCAAGGACCTGAACATGCTCGGCGCCTGCTCCGGTGGCATCACTTGTACCG

CACTGGTGGGCCACTATGCCGCCATTGGCGAGAACAAGGTCAACGCCCTGACCCTGCTGGTCAGC

GTGCTGGACACCACCATGGACAACCAGGTTGCTTTGTTTGTCGACGAGCAGACCTTGGAGGCCGC

CAAGCGCCACTCCTATCAGGCGGGCGTGCTGGAAGGCAGCGAAATGGCCAAGGTGTTCGCCTGGA

TGCGCCCCAACGACCTGATCTGGAACTACTGGGTAAACAACTACCTGCTCGGCAATGAGCCCCCC

GTGTTCGACATCCTGTTCTGGAACAACGACACCACGCGCCTGCCGGCCGCCTTCCACGGCGACCT

GATCGAAATGTTCAAGAGCAACCCGCTGACCCGCCCCGACGCCCTGGAAGTGTGCGGCACCGCGA

TCGACCTGAAACAGGTCAAATGCGACATCTACAGCCTCGCCGGCACCAACGACCACATCACCCCC

TGGCCGTCATGCTACCGCTCGGCACATCTGTTCGGCGGCAAGATCGAATTCGTACTGTCCAACAG

CGGGCATATCCAGAGCATCCTCAACCCGCCGGGCAACCCGAAGGCACGTTTCATGACCGGTGCCG

ATCGCCCGGGTGACCCGGTGGCCTGGCAGGAAAATGCCATCAAACATGCAGACTCCTGGTGGTTG

CACTGGCAGAGTTGGCTGGGCGAGCGTGCCGGCGCGCTGAAAAAGGCACCGACCCGCCTGGGCAA

CCGTACCTATGCCGCCGGCGAAGCCTCCCCAGGCACCTACGTTCACGAGCGTTGA

Figure S4. Nucleotide sequence of the *phaC1* gene with the modified nucleotides to avoid the specified restriction sites shown in Figure 9.
